# Supplementary material for: Modeling glioblastoma heterogeneity as a dynamic network of cell states
Source: Mol Syst Biol. 2021 Sep 16;17(9):e10105. doi: 10.15252/msb.202010105 (PMC8444284; doi:10.15252/msb.202010105)
Supplement: Supplementary file 5 — Source Data for Figure 3 [file MSB-17-e10105-s001.zip › Figure3A_sourcedata/GSEA_3065/nosigdb_state4.GseaPreranked.1622718430994/GAROFANO_IAVARONE_PPR_SUBTYPE.html]

Details for gene set GAROFANO\_IAVARONE\_PPR\_SUBTYPE[GSEA]

|  || Dataset | state4 |
| Phenotype | NoPhenotypeAvailable |
| Upregulated in class | na\_neg |
| GeneSet | GAROFANO\_IAVARONE\_PPR\_SUBTYPE |
| Enrichment Score (ES) | -0.7883805 |
| Normalized Enrichment Score (NES) | -3.2164555 |
| Nominal p-value | 0.0 |
| FDR q-value | 0.0 |
| FWER p-Value | 0.0 |
Table: GSEA Results Summary

  

Fig 1: Enrichment plot: GAROFANO\_IAVARONE\_PPR\_SUBTYPE      
 Profile of the Running ES Score & Positions of GeneSet Members on the Rank Ordered List

  

| PROBE | GENE SYMBOL | GENE\_TITLE | RANK IN GENE LIST | RANK METRIC SCORE | RUNNING ES | CORE ENRICHMENT || 1 | TUBA1A |  |  | 15 | 0.460 | 0.0040 | No |
| 2 | GNB1 |  |  | 523 | 0.133 | -0.0795 | No |
| 3 | SKA2 |  |  | 866 | 0.098 | -0.1357 | No |
| 4 | ZNF664 |  |  | 1196 | 0.076 | -0.1901 | No |
| 5 | GAB1 |  |  | 1209 | 0.076 | -0.1910 | No |
| 6 | TOP2B |  |  | 1236 | 0.074 | -0.1943 | No |
| 7 | CCDC88A |  |  | 1279 | 0.072 | -0.2004 | No |
| 8 | MAP2 |  |  | 1417 | 0.065 | -0.2225 | No |
| 9 | TRIB2 |  |  | 1496 | 0.062 | -0.2348 | No |
| 10 | C2orf80 |  |  | 1525 | 0.061 | -0.2386 | No |
| 11 | CDKN1B |  |  | 1650 | 0.057 | -0.2587 | No |
| 12 | IDH1 |  |  | 1691 | 0.055 | -0.2647 | No |
| 13 | PLEKHO1 |  |  | 1722 | 0.055 | -0.2689 | No |
| 14 | ZNF593 |  |  | 1882 | 0.050 | -0.2950 | No |
| 15 | PHF2 |  |  | 2008 | 0.047 | -0.3154 | No |
| 16 | CREBBP |  |  | 2250 | 0.040 | -0.3554 | No |
| 17 | KHDRBS1 |  |  | 2260 | 0.040 | -0.3564 | No |
| 18 | PDE6D |  |  | 2337 | 0.038 | -0.3686 | No |
| 19 | CDK14 |  |  | 2340 | 0.038 | -0.3684 | No |
| 20 | LRRC28 |  |  | 2530 | 0.034 | -0.3998 | No |
| 21 | WIZ |  |  | 2676 | 0.031 | -0.4238 | No |
| 22 | MEX3A |  |  | 2759 | 0.030 | -0.4372 | No |
| 23 | PPIA |  |  | 2777 | 0.029 | -0.4396 | No |
| 24 | ZSCAN21 |  |  | 2821 | 0.028 | -0.4465 | No |
| 25 | FBXO17 |  |  | 2823 | 0.028 | -0.4462 | No |
| 26 | CRTC3 |  |  | 2845 | 0.028 | -0.4494 | No |
| 27 | ARL5A |  |  | 2937 | 0.026 | -0.4643 | No |
| 28 | CUEDC1 |  |  | 3073 | 0.024 | -0.4867 | No |
| 29 | PAFAH1B3 |  |  | 3105 | 0.023 | -0.4916 | No |
| 30 | PHC1 |  |  | 3120 | 0.023 | -0.4937 | No |
| 31 | GTF2IRD1 |  |  | 3193 | 0.021 | -0.5055 | No |
| 32 | ING1 |  |  | 3266 | 0.019 | -0.5174 | No |
| 33 | KDM1A |  |  | 3324 | 0.018 | -0.5267 | No |
| 34 | VEZF1 |  |  | 3424 | 0.015 | -0.5432 | No |
| 35 | ZNF570 |  |  | 3432 | 0.014 | -0.5441 | No |
| 36 | HNRNPA0 |  |  | 3522 | 0.011 | -0.5590 | No |
| 37 | CBX5 |  |  | 3561 | 0.010 | -0.5652 | No |
| 38 | TUBA1B |  |  | 3658 | 0.007 | -0.5813 | No |
| 39 | BRD7 |  |  | 3671 | 0.006 | -0.5832 | No |
| 40 | ZNF22 |  |  | 3892 | -0.003 | -0.6203 | No |
| 41 | SPEN |  |  | 3935 | -0.006 | -0.6273 | No |
| 42 | NTRK3 |  |  | 3955 | -0.007 | -0.6304 | No |
| 43 | ACBD6 |  |  | 4105 | -0.016 | -0.6552 | No |
| 44 | GTF2IRD2 |  |  | 4137 | -0.018 | -0.6602 | No |
| 45 | RAB8A |  |  | 4161 | -0.019 | -0.6638 | No |
| 46 | TIA1 |  |  | 4162 | -0.020 | -0.6635 | No |
| 47 | SECISBP2 |  |  | 4205 | -0.022 | -0.6703 | No |
| 48 | CYTH2 |  |  | 4207 | -0.022 | -0.6701 | No |
| 49 | GABPB1 |  |  | 4231 | -0.024 | -0.6737 | No |
| 50 | MAD2L2 |  |  | 4387 | -0.035 | -0.6993 | No |
| 51 | LIN54 |  |  | 4436 | -0.039 | -0.7068 | No |
| 52 | MAML2 |  |  | 4512 | -0.045 | -0.7188 | No |
| 53 | C19orf57 |  |  | 4526 | -0.046 | -0.7203 | No |
| 54 | PHACTR4 |  |  | 4581 | -0.050 | -0.7287 | No |
| 55 | ATP9B |  |  | 4626 | -0.053 | -0.7354 | No |
| 56 | TRIM28 |  |  | 4656 | -0.056 | -0.7395 | No |
| 57 | AAAS |  |  | 4684 | -0.058 | -0.7432 | No |
| 58 | SUV39H2 |  |  | 4699 | -0.060 | -0.7447 | No |
| 59 | MAZ |  |  | 4702 | -0.060 | -0.7442 | No |
| 60 | GALE |  |  | 4750 | -0.064 | -0.7512 | No |
| 61 | ING2 |  |  | 4753 | -0.064 | -0.7506 | No |
| 62 | EXOSC2 |  |  | 4767 | -0.065 | -0.7519 | No |
| 63 | SAFB |  |  | 4792 | -0.067 | -0.7550 | No |
| 64 | DPF1 |  |  | 4795 | -0.068 | -0.7543 | No |
| 65 | TOE1 |  |  | 4802 | -0.068 | -0.7544 | No |
| 66 | TNFAIP8L1 |  |  | 4806 | -0.068 | -0.7539 | No |
| 67 | LSM6 |  |  | 4810 | -0.068 | -0.7535 | No |
| 68 | NIF3L1 |  |  | 4819 | -0.069 | -0.7538 | No |
| 69 | ZNF117 |  |  | 4835 | -0.071 | -0.7553 | No |
| 70 | RMI1 |  |  | 4837 | -0.071 | -0.7545 | No |
| 71 | POLD1 |  |  | 4853 | -0.072 | -0.7560 | No |
| 72 | ZNF704 |  |  | 4861 | -0.072 | -0.7561 | No |
| 73 | SPATA5 |  |  | 4889 | -0.074 | -0.7596 | No |
| 74 | ZNF273 |  |  | 4895 | -0.075 | -0.7594 | No |
| 75 | HNRNPR |  |  | 4924 | -0.077 | -0.7630 | No |
| 76 | CTDSPL2 |  |  | 4926 | -0.077 | -0.7621 | No |
| 77 | ARC |  |  | 4942 | -0.078 | -0.7635 | No |
| 78 | RBBP4 |  |  | 4986 | -0.082 | -0.7696 | No |
| 79 | SNRPA |  |  | 5009 | -0.083 | -0.7721 | No |
| 80 | CDK2 |  |  | 5015 | -0.084 | -0.7718 | No |
| 81 | PDE7A |  |  | 5040 | -0.086 | -0.7746 | No |
| 82 | NUP205 |  |  | 5053 | -0.087 | -0.7754 | No |
| 83 | POLA1 |  |  | 5058 | -0.088 | -0.7748 | No |
| 84 | PARP1 |  |  | 5065 | -0.088 | -0.7745 | No |
| 85 | RSBN1L |  |  | 5123 | -0.093 | -0.7828 | No |
| 86 | DSCC1 |  |  | 5133 | -0.095 | -0.7830 | No |
| 87 | SKA3 |  |  | 5136 | -0.095 | -0.7820 | No |
| 88 | PAXIP1 |  |  | 5142 | -0.096 | -0.7814 | No |
| 89 | EPB41 |  |  | 5162 | -0.098 | -0.7833 | No |
| 90 | CDC25A |  |  | 5172 | -0.099 | -0.7834 | No |
| 91 | C1orf112 |  |  | 5173 | -0.099 | -0.7819 | No |
| 92 | NSMCE4A |  |  | 5201 | -0.101 | -0.7851 | No |
| 93 | PLK4 |  |  | 5210 | -0.102 | -0.7849 | No |
| 94 | MNS1 |  |  | 5212 | -0.102 | -0.7837 | No |
| 95 | RNASEH2A |  |  | 5241 | -0.105 | -0.7869 | Yes |
| 96 | ZNF90 |  |  | 5243 | -0.105 | -0.7856 | Yes |
| 97 | CCDC138 |  |  | 5245 | -0.105 | -0.7842 | Yes |
| 98 | ELAVL1 |  |  | 5249 | -0.106 | -0.7832 | Yes |
| 99 | POP7 |  |  | 5259 | -0.107 | -0.7832 | Yes |
| 100 | DOT1L |  |  | 5264 | -0.107 | -0.7824 | Yes |
| 101 | ZNF107 |  |  | 5270 | -0.107 | -0.7817 | Yes |
| 102 | RFWD3 |  |  | 5301 | -0.109 | -0.7852 | Yes |
| 103 | DHX9 |  |  | 5302 | -0.110 | -0.7836 | Yes |
| 104 | CENPO |  |  | 5321 | -0.111 | -0.7851 | Yes |
| 105 | ZNF100 |  |  | 5339 | -0.112 | -0.7864 | Yes |
| 106 | SASS6 |  |  | 5352 | -0.114 | -0.7868 | Yes |
| 107 | C18orf54 |  |  | 5362 | -0.115 | -0.7866 | Yes |
| 108 | RCC1 |  |  | 5369 | -0.115 | -0.7860 | Yes |
| 109 | TRA2A |  |  | 5373 | -0.116 | -0.7849 | Yes |
| 110 | ZNF43 |  |  | 5375 | -0.116 | -0.7834 | Yes |
| 111 | CBX3 |  |  | 5377 | -0.116 | -0.7819 | Yes |
| 112 | H2AFV |  |  | 5381 | -0.117 | -0.7807 | Yes |
| 113 | WDR76 |  |  | 5388 | -0.117 | -0.7801 | Yes |
| 114 | SAP30 |  |  | 5390 | -0.117 | -0.7786 | Yes |
| 115 | CENPP |  |  | 5393 | -0.118 | -0.7772 | Yes |
| 116 | CKLF |  |  | 5394 | -0.118 | -0.7756 | Yes |
| 117 | KLHL23 |  |  | 5397 | -0.118 | -0.7742 | Yes |
| 118 | RECQL4 |  |  | 5399 | -0.118 | -0.7727 | Yes |
| 119 | NR2C2AP |  |  | 5400 | -0.118 | -0.7710 | Yes |
| 120 | BAZ1B |  |  | 5405 | -0.119 | -0.7700 | Yes |
| 121 | EXO1 |  |  | 5410 | -0.120 | -0.7690 | Yes |
| 122 | CCDC150 |  |  | 5426 | -0.121 | -0.7698 | Yes |
| 123 | NEIL3 |  |  | 5429 | -0.122 | -0.7684 | Yes |
| 124 | ZNF93 |  |  | 5438 | -0.123 | -0.7680 | Yes |
| 125 | RFC3 |  |  | 5450 | -0.124 | -0.7681 | Yes |
| 126 | ZNF85 |  |  | 5461 | -0.126 | -0.7680 | Yes |
| 127 | RBL1 |  |  | 5472 | -0.128 | -0.7678 | Yes |
| 128 | SKA1 |  |  | 5512 | -0.133 | -0.7725 | Yes |
| 129 | PGAP1 |  |  | 5541 | -0.135 | -0.7753 | Yes |
| 130 | GINS1 |  |  | 5542 | -0.135 | -0.7734 | Yes |
| 131 | CKAP5 |  |  | 5549 | -0.136 | -0.7724 | Yes |
| 132 | CCNF |  |  | 5562 | -0.137 | -0.7725 | Yes |
| 133 | DEPDC1B |  |  | 5570 | -0.138 | -0.7717 | Yes |
| 134 | DBF4B |  |  | 5579 | -0.140 | -0.7711 | Yes |
| 135 | HMGB3 |  |  | 5584 | -0.142 | -0.7697 | Yes |
| 136 | CENPW |  |  | 5585 | -0.142 | -0.7677 | Yes |
| 137 | DCTPP1 |  |  | 5588 | -0.143 | -0.7660 | Yes |
| 138 | NCAPH |  |  | 5595 | -0.144 | -0.7650 | Yes |
| 139 | DCLRE1C |  |  | 5596 | -0.144 | -0.7629 | Yes |
| 140 | RIF1 |  |  | 5603 | -0.145 | -0.7619 | Yes |
| 141 | CREB5 |  |  | 5611 | -0.146 | -0.7610 | Yes |
| 142 | POLA2 |  |  | 5615 | -0.146 | -0.7594 | Yes |
| 143 | POLE |  |  | 5624 | -0.147 | -0.7587 | Yes |
| 144 | CENPJ |  |  | 5625 | -0.147 | -0.7566 | Yes |
| 145 | FANCB |  |  | 5631 | -0.149 | -0.7553 | Yes |
| 146 | GEN1 |  |  | 5633 | -0.150 | -0.7533 | Yes |
| 147 | PRIM1 |  |  | 5636 | -0.150 | -0.7515 | Yes |
| 148 | HIRIP3 |  |  | 5638 | -0.151 | -0.7496 | Yes |
| 149 | CDCA8 |  |  | 5642 | -0.151 | -0.7479 | Yes |
| 150 | EXOSC9 |  |  | 5643 | -0.151 | -0.7458 | Yes |
| 151 | MCM6 |  |  | 5644 | -0.151 | -0.7436 | Yes |
| 152 | KIFC1 |  |  | 5645 | -0.152 | -0.7414 | Yes |
| 153 | CASP8AP2 |  |  | 5646 | -0.152 | -0.7393 | Yes |
| 154 | CEP152 |  |  | 5649 | -0.152 | -0.7375 | Yes |
| 155 | CEP135 |  |  | 5669 | -0.155 | -0.7385 | Yes |
| 156 | HIST2H2AC |  |  | 5670 | -0.155 | -0.7362 | Yes |
| 157 | LSM4 |  |  | 5673 | -0.155 | -0.7344 | Yes |
| 158 | DSN1 |  |  | 5687 | -0.158 | -0.7343 | Yes |
| 159 | FIGNL1 |  |  | 5699 | -0.161 | -0.7339 | Yes |
| 160 | KNTC1 |  |  | 5703 | -0.161 | -0.7321 | Yes |
| 161 | HNRNPD |  |  | 5705 | -0.161 | -0.7300 | Yes |
| 162 | MSH2 |  |  | 5711 | -0.162 | -0.7285 | Yes |
| 163 | E2F8 |  |  | 5717 | -0.163 | -0.7270 | Yes |
| 164 | ILF3 |  |  | 5722 | -0.165 | -0.7253 | Yes |
| 165 | NUCKS1 |  |  | 5724 | -0.166 | -0.7232 | Yes |
| 166 | DGCR8 |  |  | 5732 | -0.168 | -0.7219 | Yes |
| 167 | MRTO4 |  |  | 5738 | -0.169 | -0.7204 | Yes |
| 168 | GPSM2 |  |  | 5739 | -0.169 | -0.7180 | Yes |
| 169 | EXOSC8 |  |  | 5751 | -0.173 | -0.7174 | Yes |
| 170 | KIF18B |  |  | 5760 | -0.174 | -0.7162 | Yes |
| 171 | ZNF738 |  |  | 5768 | -0.177 | -0.7149 | Yes |
| 172 | BRIP1 |  |  | 5769 | -0.177 | -0.7124 | Yes |
| 173 | ZNF519 |  |  | 5775 | -0.178 | -0.7107 | Yes |
| 174 | BARD1 |  |  | 5779 | -0.179 | -0.7086 | Yes |
| 175 | MASTL |  |  | 5781 | -0.179 | -0.7062 | Yes |
| 176 | MCM2 |  |  | 5785 | -0.180 | -0.7042 | Yes |
| 177 | TRIP13 |  |  | 5792 | -0.181 | -0.7026 | Yes |
| 178 | HNRNPM |  |  | 5797 | -0.182 | -0.7007 | Yes |
| 179 | MSH6 |  |  | 5799 | -0.182 | -0.6983 | Yes |
| 180 | ZWILCH |  |  | 5805 | -0.184 | -0.6965 | Yes |
| 181 | PTBP1 |  |  | 5808 | -0.185 | -0.6942 | Yes |
| 182 | POLQ |  |  | 5818 | -0.187 | -0.6931 | Yes |
| 183 | ZNF431 |  |  | 5829 | -0.190 | -0.6920 | Yes |
| 184 | TIMELESS |  |  | 5832 | -0.190 | -0.6897 | Yes |
| 185 | BLM |  |  | 5833 | -0.191 | -0.6870 | Yes |
| 186 | PRC1 |  |  | 5835 | -0.192 | -0.6844 | Yes |
| 187 | PLK1 |  |  | 5836 | -0.192 | -0.6817 | Yes |
| 188 | FANCD2 |  |  | 5849 | -0.195 | -0.6809 | Yes |
| 189 | WDR62 |  |  | 5850 | -0.195 | -0.6781 | Yes |
| 190 | PTMA |  |  | 5852 | -0.195 | -0.6755 | Yes |
| 191 | HNRNPAB |  |  | 5862 | -0.197 | -0.6742 | Yes |
| 192 | CDCA7 |  |  | 5863 | -0.198 | -0.6714 | Yes |
| 193 | DDX11 |  |  | 5867 | -0.198 | -0.6691 | Yes |
| 194 | SRRT |  |  | 5869 | -0.199 | -0.6665 | Yes |
| 195 | CKAP2 |  |  | 5879 | -0.201 | -0.6651 | Yes |
| 196 | ZNF75A |  |  | 5883 | -0.202 | -0.6627 | Yes |
| 197 | SOX11 |  |  | 5885 | -0.203 | -0.6600 | Yes |
| 198 | SAE1 |  |  | 5888 | -0.203 | -0.6575 | Yes |
| 199 | RBMX |  |  | 5892 | -0.205 | -0.6550 | Yes |
| 200 | HAUS8 |  |  | 5897 | -0.206 | -0.6528 | Yes |
| 201 | SPAG5 |  |  | 5899 | -0.207 | -0.6500 | Yes |
| 202 | BUB1B |  |  | 5901 | -0.207 | -0.6472 | Yes |
| 203 | BRCA2 |  |  | 5910 | -0.210 | -0.6456 | Yes |
| 204 | FAM111B |  |  | 5918 | -0.211 | -0.6438 | Yes |
| 205 | LSM5 |  |  | 5932 | -0.216 | -0.6429 | Yes |
| 206 | RANBP1 |  |  | 5939 | -0.218 | -0.6408 | Yes |
| 207 | RACGAP1 |  |  | 5953 | -0.223 | -0.6398 | Yes |
| 208 | POLD3 |  |  | 5957 | -0.225 | -0.6371 | Yes |
| 209 | PKMYT1 |  |  | 5959 | -0.225 | -0.6341 | Yes |
| 210 | CENPA |  |  | 5960 | -0.226 | -0.6309 | Yes |
| 211 | NCAPD3 |  |  | 5965 | -0.227 | -0.6283 | Yes |
| 212 | NCL |  |  | 5974 | -0.228 | -0.6264 | Yes |
| 213 | DTL |  |  | 5975 | -0.229 | -0.6232 | Yes |
| 214 | RTKN2 |  |  | 5977 | -0.229 | -0.6201 | Yes |
| 215 | LMNB2 |  |  | 5978 | -0.229 | -0.6168 | Yes |
| 216 | KIF18A |  |  | 5980 | -0.230 | -0.6137 | Yes |
| 217 | MND1 |  |  | 5982 | -0.232 | -0.6106 | Yes |
| 218 | TROAP |  |  | 5983 | -0.232 | -0.6073 | Yes |
| 219 | STMN1 |  |  | 5993 | -0.236 | -0.6054 | Yes |
| 220 | KIF2C |  |  | 5996 | -0.237 | -0.6024 | Yes |
| 221 | SPC24 |  |  | 6000 | -0.238 | -0.5995 | Yes |
| 222 | LIG1 |  |  | 6002 | -0.239 | -0.5963 | Yes |
| 223 | KIF14 |  |  | 6006 | -0.240 | -0.5934 | Yes |
| 224 | HNRNPU |  |  | 6008 | -0.241 | -0.5901 | Yes |
| 225 | POC1A |  |  | 6010 | -0.241 | -0.5868 | Yes |
| 226 | SMC1A |  |  | 6017 | -0.245 | -0.5844 | Yes |
| 227 | CHEK1 |  |  | 6025 | -0.248 | -0.5820 | Yes |
| 228 | HNRNPA2B1 |  |  | 6029 | -0.251 | -0.5789 | Yes |
| 229 | CEP78 |  |  | 6031 | -0.253 | -0.5755 | Yes |
| 230 | FEN1 |  |  | 6035 | -0.256 | -0.5723 | Yes |
| 231 | DNAJC9 |  |  | 6037 | -0.260 | -0.5688 | Yes |
| 232 | SMC3 |  |  | 6041 | -0.265 | -0.5655 | Yes |
| 233 | BUB1 |  |  | 6045 | -0.266 | -0.5623 | Yes |
| 234 | HMGB1 |  |  | 6047 | -0.267 | -0.5586 | Yes |
| 235 | HJURP |  |  | 6055 | -0.271 | -0.5559 | Yes |
| 236 | CDC45 |  |  | 6063 | -0.276 | -0.5532 | Yes |
| 237 | ARHGAP11A |  |  | 6067 | -0.277 | -0.5498 | Yes |
| 238 | HNRNPH1 |  |  | 6068 | -0.277 | -0.5458 | Yes |
| 239 | MCM5 |  |  | 6069 | -0.278 | -0.5418 | Yes |
| 240 | FOXM1 |  |  | 6070 | -0.279 | -0.5379 | Yes |
| 241 | SPC25 |  |  | 6072 | -0.279 | -0.5341 | Yes |
| 242 | GMNN |  |  | 6080 | -0.285 | -0.5312 | Yes |
| 243 | KIF22 |  |  | 6089 | -0.290 | -0.5284 | Yes |
| 244 | CENPN |  |  | 6090 | -0.291 | -0.5243 | Yes |
| 245 | NUF2 |  |  | 6093 | -0.293 | -0.5205 | Yes |
| 246 | NCAPG2 |  |  | 6094 | -0.294 | -0.5163 | Yes |
| 247 | RFC2 |  |  | 6095 | -0.294 | -0.5121 | Yes |
| 248 | FANCA |  |  | 6097 | -0.295 | -0.5081 | Yes |
| 249 | CDT1 |  |  | 6098 | -0.295 | -0.5039 | Yes |
| 250 | CDCA5 |  |  | 6099 | -0.296 | -0.4997 | Yes |
| 251 | CCNA2 |  |  | 6100 | -0.297 | -0.4954 | Yes |
| 252 | CDC6 |  |  | 6103 | -0.299 | -0.4915 | Yes |
| 253 | PIF1 |  |  | 6106 | -0.301 | -0.4876 | Yes |
| 254 | DUT |  |  | 6107 | -0.303 | -0.4833 | Yes |
| 255 | CKAP2L |  |  | 6108 | -0.303 | -0.4789 | Yes |
| 256 | KIF11 |  |  | 6109 | -0.304 | -0.4746 | Yes |
| 257 | MCM10 |  |  | 6110 | -0.305 | -0.4703 | Yes |
| 258 | MELK |  |  | 6117 | -0.312 | -0.4668 | Yes |
| 259 | LBR |  |  | 6118 | -0.312 | -0.4624 | Yes |
| 260 | ASF1B |  |  | 6127 | -0.320 | -0.4592 | Yes |
| 261 | TMEM97 |  |  | 6131 | -0.324 | -0.4551 | Yes |
| 262 | LMNB1 |  |  | 6134 | -0.325 | -0.4508 | Yes |
| 263 | CDCA2 |  |  | 6135 | -0.326 | -0.4462 | Yes |
| 264 | CENPH |  |  | 6136 | -0.327 | -0.4415 | Yes |
| 265 | TACC3 |  |  | 6138 | -0.328 | -0.4370 | Yes |
| 266 | CCNB2 |  |  | 6140 | -0.329 | -0.4325 | Yes |
| 267 | NDC80 |  |  | 6142 | -0.330 | -0.4280 | Yes |
| 268 | AURKB |  |  | 6143 | -0.332 | -0.4232 | Yes |
| 269 | TK1 |  |  | 6144 | -0.334 | -0.4185 | Yes |
| 270 | NUDT1 |  |  | 6146 | -0.338 | -0.4138 | Yes |
| 271 | RRM1 |  |  | 6147 | -0.338 | -0.4090 | Yes |
| 272 | KIF15 |  |  | 6158 | -0.346 | -0.4058 | Yes |
| 273 | EZH2 |  |  | 6159 | -0.348 | -0.4008 | Yes |
| 274 | ATAD5 |  |  | 6161 | -0.350 | -0.3960 | Yes |
| 275 | KIF23 |  |  | 6162 | -0.351 | -0.3910 | Yes |
| 276 | HMGN2 |  |  | 6163 | -0.351 | -0.3860 | Yes |
| 277 | KIF4A |  |  | 6164 | -0.355 | -0.3810 | Yes |
| 278 | RAD51AP1 |  |  | 6167 | -0.357 | -0.3762 | Yes |
| 279 | ANP32E |  |  | 6168 | -0.357 | -0.3711 | Yes |
| 280 | CENPK |  |  | 6169 | -0.363 | -0.3660 | Yes |
| 281 | FUS |  |  | 6170 | -0.371 | -0.3607 | Yes |
| 282 | MCM4 |  |  | 6171 | -0.372 | -0.3554 | Yes |
| 283 | PCNA |  |  | 6173 | -0.374 | -0.3502 | Yes |
| 284 | DTYMK |  |  | 6177 | -0.381 | -0.3453 | Yes |
| 285 | ECT2 |  |  | 6178 | -0.382 | -0.3399 | Yes |
| 286 | CENPM |  |  | 6179 | -0.386 | -0.3344 | Yes |
| 287 | DBF4 |  |  | 6180 | -0.387 | -0.3289 | Yes |
| 288 | DNMT1 |  |  | 6182 | -0.396 | -0.3234 | Yes |
| 289 | CHAF1A |  |  | 6183 | -0.396 | -0.3178 | Yes |
| 290 | NASP |  |  | 6187 | -0.403 | -0.3125 | Yes |
| 291 | MYBL2 |  |  | 6190 | -0.408 | -0.3071 | Yes |
| 292 | GINS2 |  |  | 6194 | -0.420 | -0.3016 | Yes |
| 293 | ZWINT |  |  | 6195 | -0.425 | -0.2956 | Yes |
| 294 | BRCA1 |  |  | 6197 | -0.435 | -0.2895 | Yes |
| 295 | RRM2 |  |  | 6199 | -0.438 | -0.2835 | Yes |
| 296 | ESCO2 |  |  | 6206 | -0.463 | -0.2779 | Yes |
| 297 | FANCI |  |  | 6209 | -0.469 | -0.2716 | Yes |
| 298 | XRCC2 |  |  | 6212 | -0.482 | -0.2650 | Yes |
| 299 | BIRC5 |  |  | 6214 | -0.484 | -0.2583 | Yes |
| 300 | GTSE1 |  |  | 6217 | -0.490 | -0.2517 | Yes |
| 301 | CLSPN |  |  | 6218 | -0.490 | -0.2447 | Yes |
| 302 | SMC4 |  |  | 6219 | -0.490 | -0.2377 | Yes |
| 303 | HELLS |  |  | 6221 | -0.501 | -0.2308 | Yes |
| 304 | TYMS |  |  | 6222 | -0.501 | -0.2236 | Yes |
| 305 | NUSAP1 |  |  | 6223 | -0.507 | -0.2164 | Yes |
| 306 | CENPE |  |  | 6226 | -0.511 | -0.2095 | Yes |
| 307 | NCAPG |  |  | 6227 | -0.513 | -0.2022 | Yes |
| 308 | SMC2 |  |  | 6229 | -0.518 | -0.1950 | Yes |
| 309 | TMPO |  |  | 6233 | -0.522 | -0.1880 | Yes |
| 310 | CKS1B |  |  | 6234 | -0.523 | -0.1806 | Yes |
| 311 | KPNA2 |  |  | 6236 | -0.543 | -0.1730 | Yes |
| 312 | H2AFX |  |  | 6237 | -0.576 | -0.1648 | Yes |
| 313 | UBE2T |  |  | 6240 | -0.593 | -0.1567 | Yes |
| 314 | ATAD2 |  |  | 6243 | -0.607 | -0.1484 | Yes |
| 315 | UBE2S |  |  | 6244 | -0.617 | -0.1396 | Yes |
| 316 | CDK1 |  |  | 6246 | -0.627 | -0.1309 | Yes |
| 317 | MAD2L1 |  |  | 6247 | -0.628 | -0.1219 | Yes |
| 318 | HIST1H1B |  |  | 6249 | -0.646 | -0.1129 | Yes |
| 319 | H2AFZ |  |  | 6250 | -0.651 | -0.1037 | Yes |
| 320 | MCM7 |  |  | 6252 | -0.665 | -0.0944 | Yes |
| 321 | TPX2 |  |  | 6253 | -0.667 | -0.0849 | Yes |
| 322 | ASPM |  |  | 6254 | -0.680 | -0.0752 | Yes |
| 323 | UBE2C |  |  | 6255 | -0.750 | -0.0645 | Yes |
| 324 | HMGB2 |  |  | 6258 | -0.766 | -0.0539 | Yes |
| 325 | TOP2A |  |  | 6260 | -0.814 | -0.0425 | Yes |
| 326 | MKI67 |  |  | 6261 | -0.882 | -0.0300 | Yes |
| 327 | HIST1H4C |  |  | 6262 | -1.038 | -0.0152 | Yes |
| 328 | CENPF |  |  | 6263 | -1.068 | -0.0000 | Yes |
Table: GSEA details [plain text format]

  

Fig 2: GAROFANO\_IAVARONE\_PPR\_SUBTYPE: Random ES distribution      
 Gene set null distribution of ES for **GAROFANO\_IAVARONE\_PPR\_SUBTYPE**
